# Modulation of T Cell Responses by Fucoidan to Inhibit Osteogenesis

**DOI:** 10.3389/fimmu.2022.911390

**Published:** 2022-06-23

**Authors:** Hailin Huang, Fangze Guo, Xuyang Deng, Mingzhe Yan, Danyang Wang, Zhanyi Sun, Changqing Yuan, Qihui Zhou

**Affiliations:** ^1^ Department of Stomatology, The Affiliated Hospital of Qingdao University, Qingdao University, Qingdao, China; ^2^ School of Stomatology, Qingdao University, Qingdao, China; ^3^ Institute for Translational Medicine, The Affiliated Hospital of Qingdao University, Qingdao University, Qingdao, China; ^4^ State Key Laboratory of Bioactive Seaweed Substances, Qingdao Bright Moon Seaweed Group Co., Ltd., Qingdao, China; ^5^ School of Rehabilitation Sciences and Engineering, University of Health and Rehabilitation Sciences, Qingdao, China

**Keywords:** marine polysaccharide, fucoidan, T cell, osteogenesis, osteoimmunology

## Abstract

Fucoidan has sparked considerable interest in biomedical applications because of its inherent (bio)physicochemical characteristics, particularly immunomodulatory effects on macrophages, neutrophils, and natural killer cells. However, the effect of fucoidan on T cells and the following regulatory interaction on cellular function has not been reported. In this work, the effect of sterile fucoidan on the T-cell response and the subsequent modulation of osteogenesis is investigated. The physicochemical features of fucoidan treated by high-temperature autoclave sterilization are characterized by UV–visible spectroscopy, X-ray diffraction, Fourier transform infrared and nuclear magnetic resonance analysis. It is demonstrated that high-temperature autoclave treatment resulted in fucoidan depolymerization, with no change in its key bioactive groups. Further, sterile fucoidan promotes T cells proliferation and the proportion of differentiated T cells decreases with increasing concentration of fucoidan. In addition, the supernatant of T cells co-cultured with fucoidan greatly suppresses the osteogenic differentiation of MC3T3-E1 by downregulating the formation of alkaline phosphatase and calcium nodule compared with fucoidan. Therefore, our work offers new insight into the fucoidan-mediated T cell and osteoblast interplay.

## 1 Introduction

Due to their distinctive (bio)physicochemical features, such as outstanding biocompatibility, bioactivity, immunomodulation, and structural functions, marine polysaccharides have gained increasing attention in nutraceutical and biomedicine in recent decades (1–8). It is noteworthy that fucoidan is a sulfated polysaccharide extracted from brown algae with a variety of biological activities, such as immunomodulatory activity, osteogenesis, anti-inflammatory, anti-tumor, anti-bacterial, anti-viral, anti-oxidant, anti-coagulant, anti-thrombotic, and anti-fibrotic (3, 9–12). Particularly, it has a critical option for a range of biological applications due to its immunomodulatory characteristic. For instance, fucoidan prevented the development of macrophages into foam cells and the migration of smooth muscle cells into the intimal layer of the aorta, thereby inhibiting the formation of atherosclerotic plaques (10). Intraperitoneal administration of fucoidan has been reported to accelerate the recovery of leukocytes and neutrophils with activity exceeding that of recombinant human granulocyte colony-stimulating factor (11). Fucoidan also significantly restores cytotoxicity and granzyme B release levels in natural killer (NK) cells (13). Furthermore, intranasal fucoidan treatment increased the activation of dendritic cells, and NK cells, in the mediastinal lymph node (14). Fucoidan as a dietary supplement has been reported to promote the activation of tumor-infiltrating T cells and thus may be used synergistically with immune checkpoint blockade in the treatment of malignancies (15). Thus, it is critical to elicit how fucoidan modulates the immune cells, particularly T cells.

The immune system is known to be implicit in tissue repair and regeneration, where adaptive immunity, especially T cells, plays a critical role in modulating surrounding (stem) cell behavior and function/differentiation (16–21). It has been suggested that the mechanism by which T cells promote tissue repair and regeneration may be related to the control of neutrophil behavior and the regulation of inflammation (22–25). In particular, the ability of bone to heal without scar tissue formation has led to widespread interest in T cell-related bone repair and regeneration (26, 27). In studies of cyclosporine A-induced osteoporosis, it has been reported that the lack of T-lymphocyte function led to the formation of demineralized bone matrix and the loss of existing bone tissue density (28). And, it has been demonstrated that T cells had an inhibitory effect on osteoclast formation and changes in bone resorption, which is mediated by interferon- γ produced by T cells acting by interfering with the RANKL-RANK signaling pathway (29). In addition, studies on osteogenic differentiation of human mesenchymal stromal cells confirmed that media derived from CD4 T cells could increase osteogenic mineralization *in vitro* (30). Several studies have reported that T cells had an effect on osteogenesis and fucoidan had a modulating effect on a wide range of immune cells. However, the effect of fucoidan on T cells *in vitro* has not been studied. Considering the effect of T cells on osteogenesis, it is necessary to elucidate the role of fucoidan on T cells and consequently the subsequent effects on osteogenesis.

Fucoidan sterilized by high-temperature autoclave (HFD) was employed in this study to explore its influence on T cell responses. It was found that that although high-temperature autoclave treatment resulted in fucoidan depolymerization, there were no changes in its key bioactive groups. The proliferative effect of HFD on T cell proliferation increased with an increasing concentration within a certain range (0.4–50 mg/mL). On the contrary, HFD inhibited the differentiation of T cells, and the higher the concentration, the more pronounced the inhibitory effect. HFD could inhibit osteogenesis by affecting T cells. This is the first report of fucoidan being employed *in vitro* to modulate T cell responses and thereby research its effects on osteogenesis, to the best of our knowledge.

## 2 Experimental Section

### 2.1 Materials

Fucoidan [Molecular weight (Mw) = 276 kDa, purity ≥95%, sulfate: 29.65%] was provided by Qingdao Bright Moon Seaweed Group Co., Ltd. (China). EasySep™ Mouse T Cell Isolation Kit was acquired from Stemcell Technologies (Vancouver, BC, Canada). The anti-mouse CD3 (Clone 17A2) and anti-mouse CD28 (Clone 37.51) were acquired from BioGems. Annexin V-fluorescein-5-isothiocyanate (AxV), propidium iodide (PI), anti-mouse CD8α, phycoerythrin-cyanine 7 (PE-Cy7, Clone: 53-6.7), anti-mouse CD4, PE (Clone: GK1.5) and Flow cytometry Straining buffer were supplied by Multi Sciences (LianKe) Biotech,CO.,LTD. 5-(and-6)-Carboxyfluorescein diacetate, succinimidyl ester (CFSE) was provided by Abbkine Bioadvisers. RPMI 1640 and Tris-(hydroxymethyl) aminomethane (Tris) was purchased from Solarbio. Dulbecco’s modified Eagle’s medium (DMEM), fetal bovine serum and penicillin/streptomycin were acquired from Biological Industries, Israel. Cell Counting Kit-8 (CCK-8) was provided by MedChemExpress Co.Ltd (Shanghai, China). Alizarin red S (ARS) staining kit for osteogenesis was purchased from Beyotime (Shanghai, China). All the experimental water was deionized water.

### 2.2 Chemical Composition Analysis

Unsterilized fucoidan (UFD) was dried in a lyophilizer after being treated with high-temperature autoclave sterilization (121°C, 20 min). The composition of monosaccharides was determined using high performance liquid chromatography (HPLC1260, Agilent, USA). Specific experimental methods are as follows: chromatographic conditions: C column, 250mm × 4.6mm, 5hm, or equivalent; The column temperature was 40 °C The flow rate was 1.0 mL/min; Mobile phase: solvent A: acetonitrile-0.05mol/L potassium dihydrogen phosphate buffer (15 + 85), solvent B: acetonitrile-0.05mol/L potassium dihydrogen phosphate buffer (40 + 60).

### 2.3 UV–Visible Spectroscopy Analysis

2 mg of UFD and HFD were completely dissolved in 2.5 mL deionized water respectively and the spectrum was measured in the range of 200–450 nm using UV–vis spectrophotometer (UV-1800 spectrophotometer, Shimadzu, Japan).

### 2.4 X-Ray Diffraction (XRD) Analysis

XRD spectroscopy was performed using DX2700 (Dandong, China) to examine the crystal structure of fucoidan. The samples were tested between 10° and 70°, at a voltage of 40 kV, a current of 30 mA, and Cu Kα radiation (λ=1.5418 Å) at a scan rate of 0.05° (2θ)/min.

### 2.5 Nuclear Magnetic Resonance (NMR) Analysis

^1^H NMR analysis was performed using a Sophisticated Spectrometer (400 MHz Bruker model). Simply, 5 mg of UFD and HFD were dissolved in 0.55 mL of deuterium oxide and placed in NMR tubes respectively. NMR spectrum was analyzed at 27°C and proton chemical shifts were expressed in parts per million (ppm).

### 2.6 Fourier Transform Infrared (FT-IR) Analysis

The functional groups of UFD and HFD were analyzed in the FT-IR spectroscopy (Nicolet iS10, Thermo Scientific, USA). The infrared spectrum was measured in the wavenumber range of 4000-500 cm^-1^.

### 2.7 Primary T Lymphocytes Extraction and Activation

Murine cells were acquired from adult (6–12 week old) C57BL/6 mice spleens, approved by the Ethics Committee of the Affiliated Hospital of Qingdao University (approval number: QYFYWZLL 26850) (31, 32). T lymphocytes were isolated from splenocytes by negative selection using EasySep™ Mouse T Cell Isolation Kit according to the manufacturer’s instructions. Plate fixed anti-mouse CD3 (1 µg/mL) and free anti-mouse CD28 (1 µg/mL) were used overnight at 4°C to activate primary T cells. The purity of extracted T cells is tested by the anti-mouse CD3e Antibody, Clone 145-2C11 and is sufficient for subsequent experiments. T cells were cultured in the T cell culture media (RPMI 1640 supplemented with phenol sulfonphthalein, hepes, Sodium Pyruvate, L- glutamine, D-glucose, sodium bicarbonate, 10% fetal bovine serum and 1% penicillin/streptomycin), in a humidified incubator of 5% CO_2_ at 37°C.

### 2.8 Cell Proliferation

T cells were cultured in 96-well plates at a density of 1×10^6^ cells/mL, and different concentrations of HFD (0.4, 2, 10, and 50 mg/mL) were added to the medium. A simple medium without HFD was used as a control and incubated for 1, 3 and 5 d, respectively. Subsequently, 10% CCK-8 was added to the medium and incubated for 4 h at 37°C. The optical density (OD) values of the medium at 450 nm were measured using a microplate reader (SynergyH1/H1M, Bio-Tek, China). In addition to this, the cells were stained using CFSE according to the instructions of the manufacturer before culturing, and after co-culture with different concentrations of HFD for 3 d, they were washed and analyzed using a BD FACSCanto (BD Bioscience, USA).

### 2.9 Cell Apoptosis

Potential toxic effects of HFD were tested using flow cytometry. Different concentrations of HFD were used to co-culture with T cells for 2 d as described previously, also with the normal medium as a control. Cells were stained using a mixture containing 0.5 µL/mL of AxV and 0.5 µL/mL of PI and incubated for 15 minutes at room temperature protected from light. The cells were then washed twice with phosphate buffer solution and assayed using flow cytometry.

### 2.10 Cell Differentiation

For cell differentiation analysis, after spreading the plates and culturing the cells for 5 d as described previously, the cells were resuspended using flow cytometry staining buffer and stained with anti-CD4, anti-CD8, both at a concentration of 0.5 µL/mL and incubated for 15 minutes at room temperature and under protection from light. Cells were washed with flow cytometry staining buffer and resuspended again before being detected using flow cytometry.

### 2.11 MC3T3-E1 Cell Viability

The murine osteoblastic cell line (MC3T3-E1) was provided by the Cell Culture Centre of Shanghai Institutes for Biological Science Chinese Academy of Sciences (Shanghai, China). MC3T3-E1 cells were grown at a density of 5×10^4^ cells/mL in 96-well plates, DMEM medium was used as the control group (CON), while the remaining three groups were added with 20% of 0.4 mg/mL HFD supernatant (HFD), 20% of T cell supernatant (T) and 20% of supernatant from co-culture of T cells with HFD (CM), respectively. After 1 and 3 d of incubation, 10% of the Cell Counting Kit 8 (Absin Bioscience Inc., China) was added to the culture medium and incubated for 1 h at 37°C. The OD values of the medium at 450 nm were measured using a microplate reader (SynergyH1/H1M, Bio-Tek, China).

### 2.12 Alkaline Phosphatase (ALP) Staining and Activity

To obtain conditioned medium (CM), T cells were inoculated into 6-well plates at a density of 1× 10^6^ cells/mL and co-cultured with 0.4 mg/mL HFD. Cell suspensions were collected after 24 h, centrifuged, and the supernatants were collected and stored in a -80°C freezer until use. For ALP staining, MC3T3-E1 cells were inoculated at a density of 2 × 10^4^ cells per well in 24-well plates and treated with osteogenic induction medium (0.1 mM dexamethasone,10 mM β-glycerophosphate and 50 µg/mL L-ascorbic acid in basic DMEM medium) containing CM at 20% concentration. The osteogenic induction medium alone group (CON), the group containing 20% 0.4 mg/mL HFD supernatant (HFD) and the group containing 20% T cell supernatant (T) were used as controls, and HFD, T, and CM were added at each medium change (33). Following the manufacturer’s instructions, the ALP activity of MC3T3-E1 cells was determined using an ALP assay kit (Nanjing Jiancheng Bioengineering Institute, Nanjing, China) after 14 days of incubation. Inverted Fluorescence Microscopy (Nikon A1 MP, Japan) was used to capture the photos. The ALP staining positive rate was quantified using ImageJ software. For standardization, total cellular protein concentration was determined by the BCA protein assay kit (Beyotime, Shanghai, China). ALP activity was shown as total cellular protein content (king unit/g prot). The ALP staining positive rate was quantified using ImageJ software. For standardization, total cellular protein concentration was determined by the BCA protein assay kit (Beyotime, Shanghai, China). ALP activity was shown as total cellular protein content (king unit/g prot).

### 2.13 ARS Staining

Calcium accumulation was determined by ARS staining. MC3T3-E1 cells were treated and cultured in the same way as before. After 14 d of incubation, the cells were treated with 70% (v/v) ice-cold ethanol for 1 h at 4°C, stained with 40 mM ARS solution, and imaged using a microscope.

### 2.14 Statistical Analysis

All data points were displayed as mean values ± standard deviation (SD). Graphpad Prism 8 or Origin 9 were used to evaluate all statistical analysis. To determine differences between groups, a one-way analysis of variance (ANOVA) with Tukey’s test was used. Statistical significance was defined as a value of *p* < 0.05.

## 3 Results and Discussion

### 3.1 Physicochemical Characterization of Fucoidan

In general, biomaterial sterilization is an essential part of microbial eradication in biomedical applications. Because of its capacity to remove nanoscale bacteria (e.g., virus and mycoplasma), a high-temperature autoclave was used to sterilize the aqueous solution of fucoidan in this situation.

The impact of a high-temperature autoclave on the Mw of fucoidan was examined using HPLC, as indicated in **Table 1**. The chemical composition of HFD remained unchanged, however, the Mw and the ratio of the components changed. The result shows that the MW of the HFD (14 kDa) was significantly lower than that of the UFD (223 kDa), indicating that the high-temperature autoclave treatment led to the depolymerization of the fucoidan. Studies have reported that low-molecular-weight fucoidan has higher absorption and bioavailability than high-molecular fucoidan, so autoclaved fucoidan may be more beneficial for our subsequent cellular experiments (7). The total sugar content of UFD was 78.61%, sulfate group content was 28.42%, and uronic acid content was 0.9%. Compared with UFD, the total sugar content of HFD decreased significantly, sulfate group content increased slightly and uronic acid content increased greatly.

**Table 1:** The Mw and chemical composition of UFD and HFD.

Samples	Mw (kDa)	Total sugar (%)	Sulfate (%)	Uronic acid (%)
**UFD**	223	78.61	28.42	0.9
**HFD**	14	70.91	30.23	10.7

**Figure 1:**
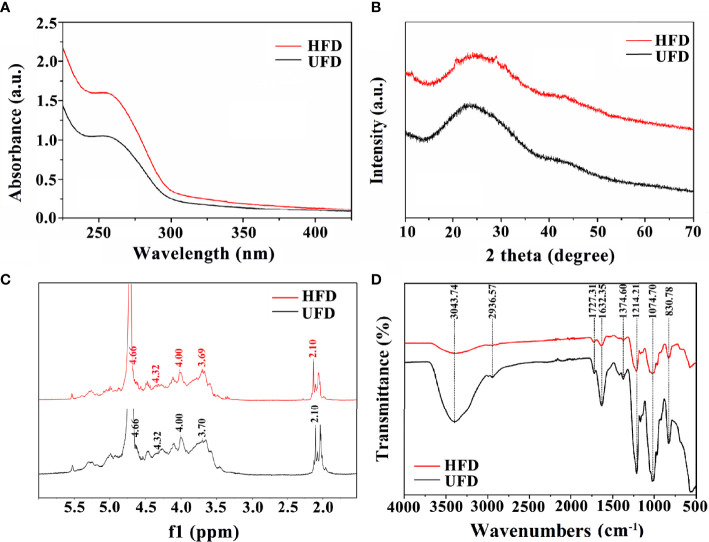
**(A)** UV-visible spectroscopy, **(B)** XRD diffraction patterns, **(C)** ^1^HNMR spectrum, and **(D)** FT-IR spectra of UFD and HFD.

UV–visible spectroscopy, XRD, ^1^HNMR, and FT-IR measurements were used to further evaluate the physicochemical characterizations of fucoidan. Both UFD and HFD appeared the typical absorbance peak at 260 nm within the UV–visible which indicates the covalent holding of aromatic compounds with the polysaccharides (**Figure 1A**). Additionally, the absorption band of fucoidan suggested fucose enriched sulfated polysaccharides (34). The changes in microstructure were determined by XRD analysis. As shown in **Figure 1B**, both the UFD and HFD powder at 23° showed a low overall crystallinity, which indicates that it could be a semicrystalline polymer, in line with previous research findings (35, 36).. In addition, NMR spectroscopy has served as a useful tool for studying the anomeric configuration and sulphation modes of polysaccharides. In this study, the ^1^HNMR spectrum of UFD and HFD is shown in **Figure 1C**. Signals at 4.32 and 4.66 ppm corresponded to α-L-fucose and 3-linked α-L-fucose, which were the primary components of fucoidan (34, 37). The 3-linked β-D-galactose was represented by the signal at 4.00 ppm. The presence of α-L-rhamnopyranosyl residues was suggested by the signal at 3.70 ppm (38). FT-IR was used to investigate the influence of high-temperature autoclave sterilization on the chemical group of fucoidan. The key characteristic peaks (3440, 2937, 1640, 1250, 1053, and 840 cm^-1^) were found in the FT-IR spectra of UFD and HFD, as shown in **Figure 1D**. The O**-**H group stretching vibrations related to a large peak between 3600 and 3000 cm^-1^. Signals at 2936 and 1632 cm-1 were ascribed to C**-**H and C**═**O stretching vibration, and the band at 2936 cm^-1^ was recognized as the typical absorption of polysaccharides (39, 40). The band at 1250 cm^-1^ was considered to correlate to the S**═**O stretching vibration, which was regarded as the most prominent feature of fucoidan, indicating the existence of sulfate groups. The peak at 832 cm^-1^ was attributed to the C-O-S bending vibration, which indicated sulfate groups at the axial C-4 position of fucose (3, 34). Taken together, these results indicate that although high-temperature autoclave treatment resulted in fucoidan depolymerization, there were no changes in its key bioactive groups.

### 3.2 The Interactions Between HFD and T Lymphocytes

**Figure 2:**
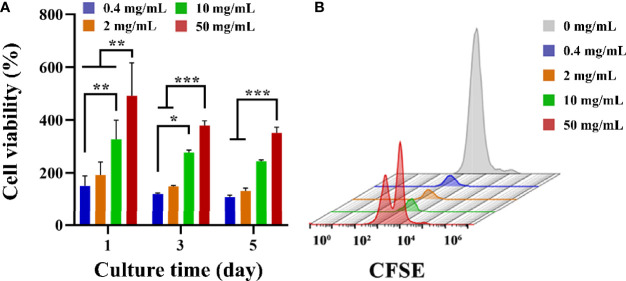
**(A)** T cell viability on different groups treated with HFD after 1, 3 and 5 d. Data are mean ± SD (n = 3) (*p < 0.05, **p < 0.01, ***p < 0.001). **(B)** Representative CFSE diagram on various samples.

CCK-8 was used to detect the effect of fucoidan on T cell viability. CCK-8 viability was measured after 1, 3 and 5 d of co-cultured with HFD. As presented in **Figure 2A**, on the first day, the addition of HFD resulted in a significant increase in cell viability, and the higher the concentration, the more significant the increase in cell viability. On days 3 and 5, the addition of HFD still had an increasing effect on cell viability, and the higher concentration groups (i.e., 10, and 50 mg/mL) still greatly increased cell viability. Furthermore, T cell proliferation was assessed by flow cytometry and CFSE staining. CFSE was chosen because the two acetate groups made it more cell-permeable and non-fluorescent than CFSE (41). Intracellular esterase cleaved the acetate groups of CFSE, resulting in the luminous, non-cell permeable CFSE. This dye was uniformly distributed between the two daughter cells during each cell division, resulting in a 50% reduction in CFSE fluorescence intensity with each cell division. The proliferation of the cells could be tracked in this way, and the flow cytometry results for 3 d following stimulation are presented in **Figure 2B**. The HFD group at 50 mg/mL showed a double peak, proving that this group had the best proliferation-promoting effect. Collectively, these data demonstrated that HFD greatly enhanced the growth of T cells.

**Figure 3:**
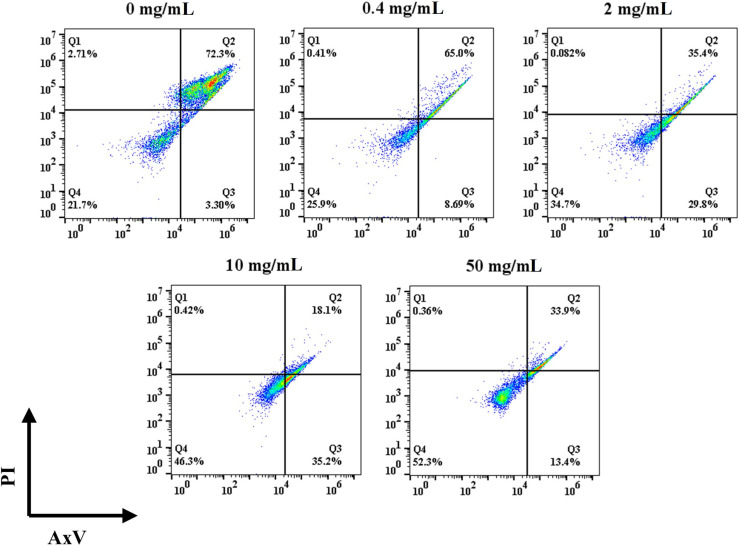
Effect of HFD on the viability of mouse primary T cells.

Apoptotic and necrotic cells were detected with AxV and PI. As shown in **Figure 3**, the percentage of viable cells in the control group was 19.8% after 3 d of incubation. The addition of HFD increased the percentage of viable cells, and it gradually increased with the increase of HFD concentration. In details, the percentages of viable cells at 0.4, 2, 10, and 50 mg/mL of HFD were 25.9%, 30.6%, 46.1% and 52.6%, respectively. In addition, the addition of HFD resulted in a decrease in the ratio of late apoptotic to necrotic cells. To be specific, the ratios of late apoptotic and necrotic cells in the control, 0.4, 2, 10, and 50 mg/mL fucoidan were 75.4%, 65.2%, 38.3%, 18.8% and 27.2% respectively. The low overall percentage of live cells may be since IL-2 is not added to the cultures as it has been reported that T cells themselves can produce IL-2 to participate in certain feedback loops (42–45). Therefore, our results demonstrate that HFD reduces T cell apoptosis even in the absence of IL-2.

**Figure 4:**
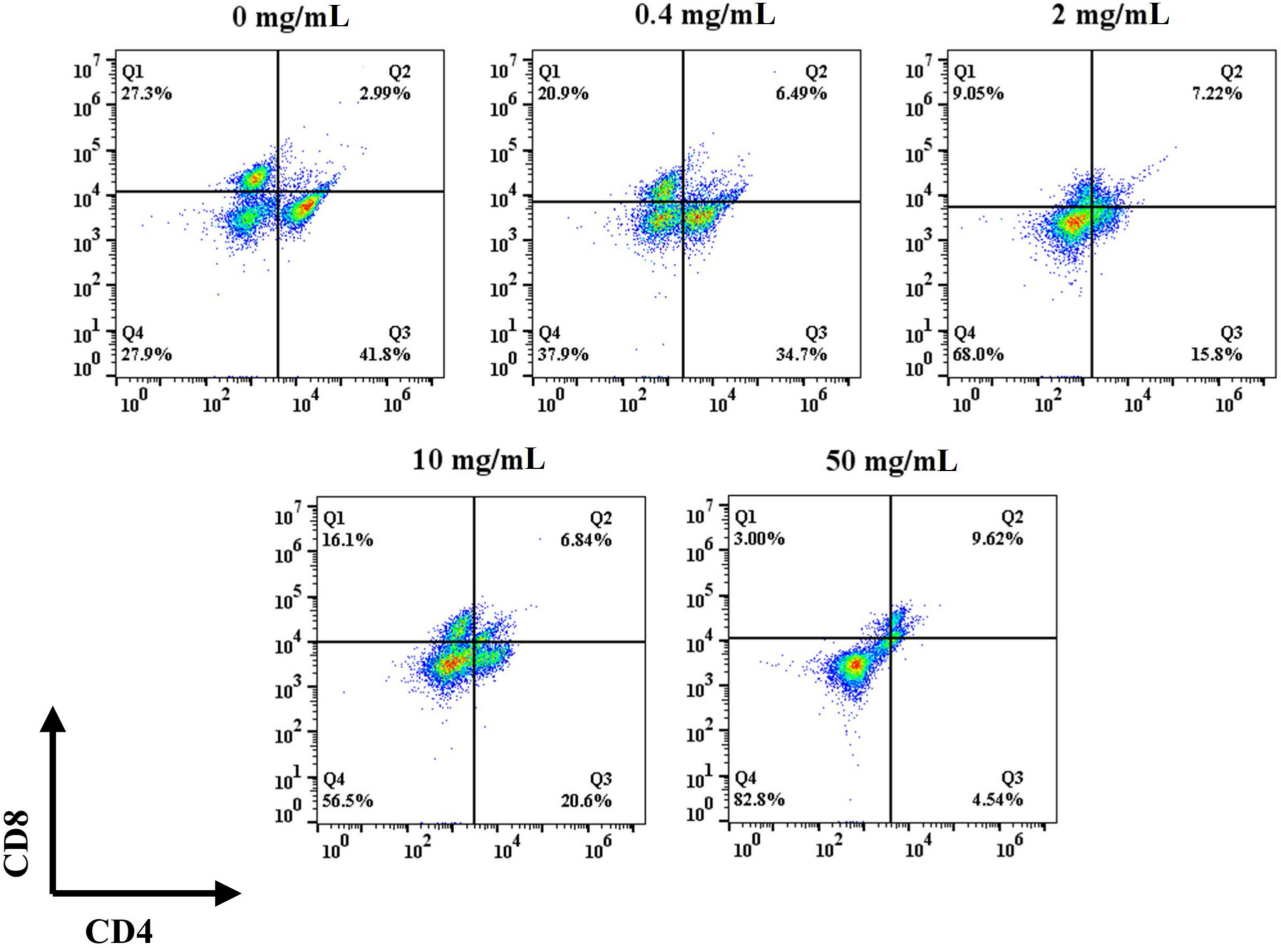
Effect of HFD on the mouse primary T cells differentiation.

Differentiation experiments were performed on day 5 after seeding to evaluate the phenotype of the T cells after proliferation. Specifically, naïve T (CD4^-^/CD8^-^), CD4^+^, and CD8^+^ T cells were identified by flow cytometry. In the control group, the naïve T cells ratio was 27.9% and the ratio of CD4^+^ to CD8^+^ cells was 1.53 (**Figure 4**). The ratio of CD4^+^ to CD8^+^ did not show a trend with concentration after the addition of HFD, but the ratio of initial T cells gradually increased with the increase of HFD addition, with the ratios of 37.9%, 68%, 56.5%, and 82.0%, respectively. The 0.4 mg/mL HFD group had the greatest effect on T cell differentiation, and the ratio of CD4^+^ to CD8^+^ cells was 1.66. Differentiated T cells in this group may produce more cytokines, which may have the greatest effect on subsequent osteogenic differentiation.

### 3.3 MC3T3-E1 Cell Viability and Osteogenic Differentiation

**Figure 5:**
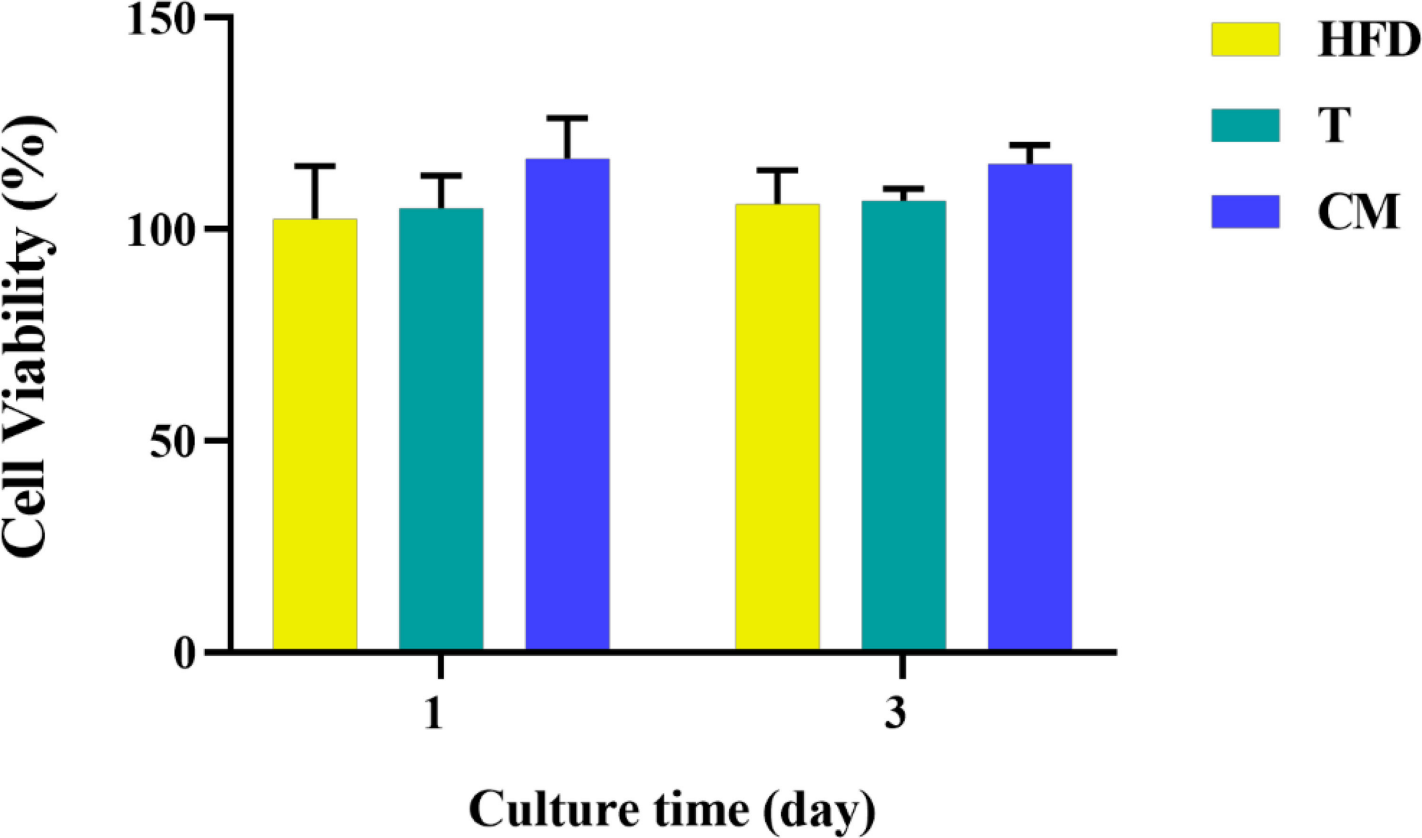
MC3T3-E1 cell viability on various samples for 1 and 3 d. HFD: the group containing HFD; T: the group containing T cell supernatant; CM: supernatant from co-culture of T cells with HFD.

Based on the differentiation of T cells above, 0.4 mg/mL HFD was chosen for the following experiments. In conjunction with studies by others, supernatants from one day after co-culture of HFD with T cells were used as CM (33). After 1 and 3 d of culture, we examined the effect of CM on MC3T3-E1 cell activity by CCK-8. As shown in **Figure 5**, MC3T3-E1 cell activity increased significantly with time in all groups. However, compared with the CON, the CM group did not significantly increase the viability of MC3T3-E1 cells. Therefore, HFD did not contribute to the viability of MC3T3-E1 cells by regulating T cells.

**Figure 6:**
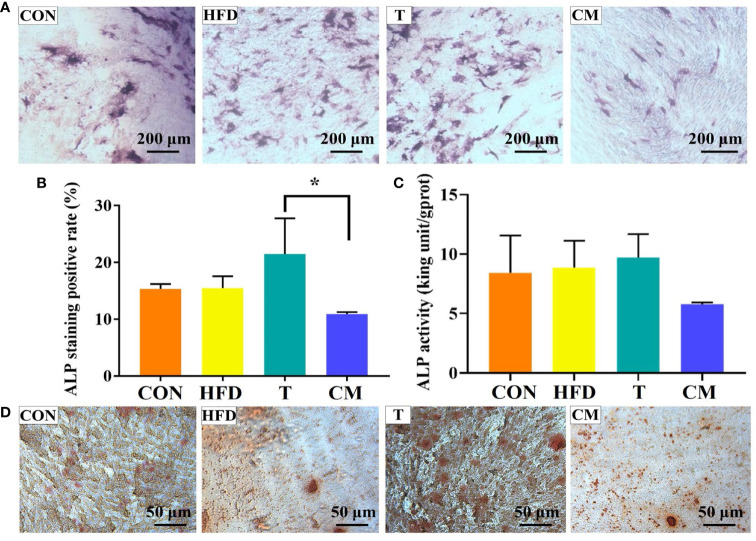
**(A)** ALP activity of MC3T3-E1 cells on different groups after 14 d of culture. **(B)** ImageJ was used to quantify the ALP staining results (*p < 0.05). **(C)** ALP test after 14 d for each group using the ALP assay kit. **(D)** The mineralized nodules were stained with ARS after 14 d.

ALP has been regarded as an important biochemical marker for osteogenic differentiation and new bone production (46–49). **Figure 6A** represented microscopic photographs of cellular ALP expression in CON, HFD, T, and CM after 14 d of culture. It was found that the area of ALP staining was larger in the HFD and T compared with the CON, while the area of ALP staining was significantly reduced in the CM compared to other groups. To better understand the ALP staining results, we quantified the stained area (**Figure 6B**). Quantitative analysis of ALP staining also confirmed these results above. HFD or T alone slightly promoted the expression of ALP compared with the CON, while CM caused a decrease in ALP expression. **Figure 6C** presented the cellular ALP activities of each group using the ALP assay kit. ALP expression decreased in the CM group compared with other groups. Further, ARS, a dye that binds to Ca ions, was used to color mineralized nodules generated by extracellular matrix Ca deposition (50). As shown in **Figure 6D**, in all groups, the ARS staining revealed a brilliant red color of calcified nodules. The area stained with ARS was less in the CM group compared with the control group. Taken together, our results demonstrate that HFD could inhibit the osteogenic differentiation of MC3T3-E1 cells by regulating T cells.

## 4 Conclusions

In summary, high-temperature autoclave sterilization had no negative effect on the bioactive group of UFD. The molecular weight of UFD was reduced due to depolymerization. HFD modulated the proliferation and differentiation of T cells, and the proliferative effect of HFD on T cell proliferation increased with an increasing concentration within a certain range (0.4–50 mg/mL). On the contrary, HFD inhibited the differentiation of T cells, and the higher the concentration, the more pronounced the inhibitory effect. HFD could inhibit osteogenesis by affecting T cells. Thus, our findings provide new insight into the fucoidan-mediated T cells and the following regulatory on osteogenesis.

## Data Availability Statement

The original contributions presented in the study are included in the article/Supplementary Material. Further inquiries can be directed to the corresponding author.

## Ethics Statement

The animal study was reviewed and approved by The Ethics Committee of the Affiliated Hospital of Qingdao University.

## Author Contributions

QZ and CY contributed to conception and design of the study. HH, FG, XD, DW, and ZS performed the experiments. HH, MY and CY performed the statistical analysis. HH, CY, and FG wrote the first draft of the manuscript. QZ reviewed the draft of the manuscript. QZ and CY provided the funding. All authors contributed to manuscript revision, read, and approved the submitted version.

## Funding

The authors are very grateful for the financial support by National Natural Science Foundation of China (Grant No. 31900957), Shandong Provincial Natural Science Foundation (Grant No. ZR2019QC007), Innovation and technology program for the excellent youth scholars of higher education of Shandong province (Grant No. 2019KJE015), and Traditional Chinese Medicine Science and Technology Project of Shandong province (Grant No. 2021Q069).

## Conflict of Interest

Author ZS is employed by Qingdao Bright Moon Seaweed Group Co., Ltd.

The remaining authors declare that the research was conducted in the absence of any commercial or financial relationships that could be construed as a potential conflict of interest.

## Publisher’s Note

All claims expressed in this article are solely those of the authors and do not necessarily represent those of their affiliated organizations, or those of the publisher, the editors and the reviewers. Any product that may be evaluated in this article, or claim that may be made by its manufacturer, is not guaranteed or endorsed by the publisher.

## Abbreviations

NK, natural killer; HFD, Fucoidan sterilized by high-temperature autoclave; Mw, Molecular weight; AxV, Annexin V-fluorescein-5-isothiocyanate; PI, propidium iodide; CFSE, 5-(and-6)-Carboxyfluorescein diacetate, succinimidyl ester; DMEM, Dulbecco’s modified Eagle’s medium; CCK8, Cell Counting Kit-8; ARS, Alizarin red S; UFD, unsterilized fucoidan; HPLC, high performance liquid chromatography; XRD, X- ray diffraction; NMR, Nuclear magnetic resonance; FT-IR, Fourier transform-infrared spectroscopy; OD, The optical density; MC3T3-E1, The murine osteoblastic cell line; ALP, Alkaline phosphatase.
